# Clinical directors’ views of centralisation and commissioning of cleft services in the UK

**DOI:** 10.1186/1472-6831-15-12

**Published:** 2015-01-22

**Authors:** Aidan Searle, Julia K Scott, Jonathan Sandy, Andrew Ness, Andrea Waylen

**Affiliations:** School of Oral and Dental Science, Lifecourse Epidemiology and Population Oral Health, University of Bristol, Lower Maudlin Street, Bristol, BS1 2LY UK; Department of Orthodontics, Derriford Hospital, Plymouth, PL6 8DH UK; School of Oral and Dental Sciences, Faculty of Medicine and Dentistry, University of Bristol, Lower Maudlin St, Bristol, BS1 2LY UK; Bristol Nutrition Biomedical Research Unit, UHBristol Education Centre, Lower Maudlin Street, Bristol, BS1 2LY UK

**Keywords:** Specialist services, Centralisation, Commissioning, Clinical directors

## Abstract

**Background:**

To determine the views of Clinical Directors working in the United Kingdom (U.K.) Cleft Service with regard to centralisation, commissioning and impact on cleft service provision.

**Methods:**

In-depth qualitative interviews were conducted with 11 Clinical Directors representing regional cleft services. Interviews were transcribed verbatim, a coding frame was developed by two researchers and transcripts were coded using a thematic, ‘interpretive’ approach.

**Results:**

Clinical Directors perceived the commissioning of cleft services in the U.K. to be dependent upon historical agreements and individual negotiation despite service centralisation. Furthermore, Clinical Directors perceived unfairness in the commissioning and funding of cleft services and reported inconsistencies in funding models and service costs that have implications for delivering an equitable cleft service with an effective Multidisciplinary Team.

**Conclusions:**

National Health Service (NHS) commissioning bodies can learn lessons from the centralisation of cleft care. Clinical Directors’ accounts of their relationships with specialist commissioning bodies and their perspectives of funding cleft services may serve to increase parity and improve the commissioning of cleft services in the U.K.

## Background

### Centralisation of cleft services

Cleft lip and/or palate affects around one in 700 newborn babies and is the most common craniofacial anomaly [1]. Providing care for a child born with cleft lip and/or palate can be complex and may require a wide range of treatments from birth to early adulthood. The aim of treatment is to restore anatomy (face and dentition) and function (hearing, speech and feeding) and to encourage the physical and psychological development of the child.

The 1998 Clinical Standards Advisory Group (CSAG) reported that care for cleft lip and/or palate was, at that time, provided by 76 surgeons in 57 centres in the United Kingdom (UK) [2]. Treatment outcomes from some U.K. Cleft Service centres were less satisfactory than those elsewhere in Europe and a series of recommendations were made with the aim of improving child health outcomes, satisfaction with services and psychosocial development. These included centralisation of cleft services and care provision in the U.K. through the development of Multidisciplinary Teams (MDTs). There are also potential benefits of service provision via Multidisciplinary Teams e.g. training under consultant supervision in a centralised system means that expertise can be shared directly with clinical colleagues. Similarly, the sharing of good practice such as critical incident reporting, development of guidelines and also audit and research processes can inform and benefit the delivery of care [3]. The CSAG report proposed a centralised ‘hub and spoke’ model of care comprising central ‘hub’ units working with a series of peripheral ‘spoke’ units. Highly specialised inpatient and outpatient care is delivered in hubs whilst spoke units facilitate delivery of certain aspects of care conveniently closer to family homes. Around 15 years later, a national survey concluded that U.K. Cleft Services have been restructured to eleven centralised services with seventeen primary operative sites and 61 peripheral sites [4]. All services provide care through an Multidisciplinary Team model with specialist health professionals such as orthodontists, speech and language therapists, audiologists and psychologists working together with their surgical colleagues [2] although the actual composition of each team varies across sites [4].

### Commissioning of cleft services

In April 1999, as cleft services were being centralised, Primary Care Trusts were established in England and to commission health services. This role entailed assessing local health needs, planning and securing health services and improving health within the framework of NHS standards. Commissioning also requires continued accountability to the Secretary of State and adherence to the financial duties stipulated in the National Health Service (NHS) Act 1999 [5]. Commissioners have responsibility for facilitating the care of children born with cleft lip and/or palate in England and Wales and are guided by the Cleft Development Group. This independent body represents patients, clinicians and commissioners and is responsible for the CRANE database. The CRANE database contains clinical information about adults and children born with cleft lip and/or cleft palate throughout England and Wales and records information on all U.K. Cleft Service procedures. Contributions from Primary Care Trust budgets fund day to day cleft services and highly specialised cleft care services via a national budget. Specialist healthcare activities are subject to collaborative commissioning arrangements and are commissioned by a Specialised Commissioning Group. These specialist healthcare activities are identified in the National Definition Set published by the Department of Health [6]. The National Definition Set was intended to provide a basis for service reviews and strategic planning, enabling commissioners to compare activity and spending between centres. Specialised health service commissioning was intended to establish a single national function with consistency, equity and excellence at its core. Furthermore, it was intended that those seeking cleft care services would have equal access to high quality, expert care and improved health outcomes.

In 2006 the Carter review [7] reported that the National Definition Set was too rigid in defining specialised services and recommended its revision to enable wider support amongst commissioners [8]. Much of the debate focused on how well commissioning had (or had not) been undertaken by Primary Care Trusts. At the time of the current study the NHS was organised into ten Strategic Health Authorities each with Primary Care Trusts and Specialised Commissioning Groups. Clinical Directors from cleft teams negotiated with Specialised Commissioning Group commissioners for funding and provision of cleft services. The Clinical Directors were responsible for budgets regarding surgical procedures, staff recruitment, management and training and other peripheral aspects of service delivery. There were local variations in care [7] as Primary Care Trusts could disengage from specialised commissioning albeit with an increased risk of reduced funds in periods of financial restraint and consequent diminished services. This ‘shared responsibility’ for specialised commissioning increased the potential for poor regulation and performance management and lack of accountability [8].

Since the present research was undertaken, commissioning Primary Care Trusts have ceased to exist and the specialised commissioning function has been transferred to Clinical Reference Groups that cover all prescribed specialised services. To our knowledge, there is no research examining Clinical Directors’ perspectives on the commissioning of cleft care services and consequent impact on service delivery since centralisation. Thus Clinical Directors from the U.K. Cleft Service were invited to take part in this qualitative case study as part of the national survey [4] in order to investigate their views of service centralisation, commissioning and provision of multidisciplinary cleft care.

## Method

This study was nested within a national survey of U.K. Cleft Services including 11 cleft services consisting of 17 primary operative sites (some services have two primary operative sites) and 61 peripheral sites [4]. In two separate instances, teams working at two operative sites considered themselves as a unified team with a single Clinical Director resulting in 15 individual teams. Two teams working at independent operative sites were supervised by one Clinical Director so that there are 15 cleft teams and 14 Clinical Directors.

The aim of the research was to investigate Clinical Directors’ views of cleft service centralisation, commissioning and provision of multidisciplinary cleft care in the U.K.

The study design was reviewed by National Research Ethics Service (NRES) and as it was a service survey it was deemed not to require ethical approval [9].

### Clinical directors

All Clinical Directors from the 14 U.K. cleft teams attending were invited to attend a research workshop in (March 2010, where the study was introduced). After that J.K.S sent each Clinical Director a letter and invited them and their teams to participate in the study.

Eleven of the 14 Clinical Directors consented to be interviewed following the collection of quantitative survey data about the provision of care in each U.K. Cleft Service. However, three of the 14 Clinical Directors did not respond to the request for an interview despite reminder emails sent by J.K.S. Therefore unfortunately their reasons for non-participation are not known. Where Clinical Directors agreed to take part in the interviews, J.K.S. also sent out the interview schedule at least two weeks in advance so all had an opportunity to prepare for the interview.

The Clinical Directors had worked in cleft services for varying amounts of time but no more than 20 years. Their clinical specialties included plastic surgery, maxillofacial surgery, orthodontics and speech & language therapy. The Clinical Directors had worked in cleft services for varying amounts of time but no more than 20 years. Five of the 11 Clinical Directors were female.

Interviews followed a semi-structured topic guide that directed the discussion and allowed participants to elaborate on specific aspects of cleft care relevant to them. Topics for discussion included Multidisciplinary Team working, location of services, service provision, funding, interactions and relationships with other multidisciplinary professionals, users, carers, voluntary organisations, and commissioners.

All interviews were conducted by J.K.S. between April 2010 and June 2012. Seven were conducted face-to-face in a location of the Clinical Directors’ choice and four were conducted via telephone. All interviews were recorded with a digital voice recorder following verbal consent. All were transcribed verbatim, one by J.K.S and the rest by an independent transcriber. All transcripts were verified for accuracy by J.K.S against the original interview recording and emailed to each interviewee for approval. Random study numbers were allocated to each transcript at the start of the study in order to maintain anonymity before analysis by A.S.

### Data analysis

Transcripts were read and reread by A.S. and J.K.S. to gain familiarity with the data. A.S. and JS met to discuss the transcripts using an ‘interpretative’ approach through which each was aware that interpretation of the interview content may have been influenced by their own perspectives and values [10]. The analysis was inductive such that emerging themes were identified independently by A.S. and J.K.S. and then verified through discussion between them.

The coding frame was based on these themes and four transcripts were independently manually coded by both researchers who then met to compare and resolve discrepancies in coding. An overlap was apparent between some codes therefore a more definitive coding frame was developed and further codes identified. Despite these efforts, some data remained pertinent to more than a single code and have been coded accordingly.

Transcripts were electronically coded by A.S. using NVivo version 9 (2010) [11]. Data were summarised as sub-themes using a framework approach and emerging themes were verified by A.S. and J.K.S. for consistency and comprehensiveness [12]. The framework also helped to identify participants’ views in relation to specific themes and for comparison between participants.

## Results

### Themes

The analysis and coding process led to the identification of two key themes: ‘Centralisation and historical context’ and ‘Funding and Resources’. The sub-themes for each are shown in Table 1. However, the findings pertaining to the key themes and sub-themes are presented together as these themes were not considered to be mutually exclusive concepts and to provide context. For example, it became clear that ‘Commissioning and Funding’ was contingent with ‘Relationship with Commissioners’; and ‘Impact on Staff’ was contingent with ‘Staffing Levels’.

**Table 1:** **Themes and subthemes identified in the analysis**

Key theme	Centralisation and historical context	Funding and resources
**Subthemes**	• Pre-CSAG service	• Relationship with commissioners
	• Post-CSAG service	• Funding model/rationale
	• Changes to service	• Budget allocation
	• Consultation and negotiation	• Staffing levels
	• Commissioning and funding new services	
	• Capacity	
	• Impact on staff	

### Pre-CSAG service

Many Clinical Directors recalled that, before centralisation, cleft care units often worked together in a way that was similar to the way in which Multidisciplinary Teams (MDT) were presently working in the U.K. Cleft Service:

*“Pre-CSAG everyone was pretty much working together as an MDT anyhow”.* (CD 9)

They also believed that one of the positive outcomes of the CSAG report was that it had helped to facilitate relationships within their teams:

*“CSAG has facilitated a lot of working really … we’ve done team building – it’s been quite positive for people to feel part of a big team but small enough that nobody loses their identity within it”.* (CD 10)

Some Clinical Directors knew that changes were imminent and tried to work towards a Multidisciplinary Team model before the CSAG report was published:

*“… we tried to make changes independently based on the recommendations of the CSAG report. And we got absolutely nowhere. We had a series of meetings going with commissioners from several of the Health Authorities. And even though there was a lot of sympathy and the meetings continued we still never actually achieved anything. Not one thing was achieved during that period.”* (CD 2)

Prior to CSAG, consultation was undertaken with both cleft care services and the public as specialist commissioners were unable to decide whether certain teams should have single or multiple site surgery:

*“The decision was made as a result of a public consultation that ran for four months …It took a year after that for the two lead trusts to agree on the mechanism of how the service would run. What was agreed was that the Trusts between them would advertise for the post of Clinical Director then they would make the decision that the other Trust would be the lead Trust for the network.”* (CD 4)

### Post CSAG service

The post-CSAG delivery of cleft services was contingent on funding and budget allocation from the Primary Care Trusts but this process was not always transparent. While some teams received funds based on an historical budget that increased annually, others received ‘central’ funding for the lead clinician, Clinical Director and the coordination of the team. Specialist services such as audiology and orthodontics were funded locally.

However, some Clinical Directors appeared to be unclear about the commissioning and funding of services:

*“It used to be when they were doing their annual business cases we would have an awayday workshop, a lot of them don’t know where the money comes from or how much money they have coming in or out. How much they spend – it’s all a mystery”?* (CD 3)

### Changes to service/negotiation and consultation

While some Clinical Directors appointed staff to comply with the Multidisciplinary Team model of care recommended in the CSAG report [1] representation of team members at Multidisciplinary Team clinics was perceived to be inconsistent across centres [4]. In part, this seemed to be a function of funding arrangements, the perceived needs of the Clinical Director and the historical and geographical context of each cleft centre. Some teams wrote business plans in the years following CSAG to make the case for more team members and priorities were decided as money came in to the service:

*“We’ve got more team members now since we had the business case funded – we’ve got the specialist nurse, clinical psychologist which was after we had formed as a network. Prior to that we had two speech therapists a surgeon and orthodontist.”* (CD 9)

Following centralisation, Clinical Directors sometimes reported that it was difficult to find a balance given the speed of change within the Cleft Service.

*“You’ll get some people who want it to all stay the same. Never want it to change, didn’t believe in the vision of going to a centralised system. We haven’t gone quick enough for some people. In 2004 the Strategic Health Authorities told us you have to maintain the status quo, and they said “if you go and try and close all the peripheral clinics down you will have to go out to public consultation, you’ll have to carry the parental group with you” etc. etc. etc. And so we’re not moving quick enough for some people, we’re moving too quickly for another”.* (CD 1)

Despite such uncertainties, this team was able to develop a patient-centric service for the Cleft Treatment Pathway:

*“You never had any structure …it was individual surgeon-centric according to what they fancied doing, utilising their different specialist services as they had it before. Now it’s team-centric I like to think, so in other words its patient-centric with a team rotating round the patient as opposed to the patients and the other staff rotating around the surgeon and that definitely has changed.* (CD 1)

### Commissioning and funding/relationship with commissioners

Funding arrangements were determined by relationships with commissioners. Successful applications by Clinical Directors secured the finances required to deliver their service. Clinical Directors undertook and encouraged a pro-active approach with regard to identifying and developing good relationships with their commissioners in order to build a strong rapport with them and potentially optimise funding opportunities:

*“We have a formal meeting every year. Which I think is terribly important and I would encourage every unit to do that, so you know who that individual is and they need to be able to pick up the phone and speak to them.”* (CD 7)

Unfortunately, this wasn’t always the case and sometimes remote relationships with commissioners were seen as an obstacle:

*“Relationships with commissioners are very distant. I mean I’ve struggled to get to speak to commissioners. We put together this bid which went to the commissioners and I did get a chance to speak to somebody on the specialised commissioning team about the bid but that was once or twice after the 3 years maybe. And since then really the commissioners have not asked us anything. I have tried very hard to speak to them because I’ve been trying to argue for more resource …”* (CD 8)

Some Clinical Directors were concerned that commissioners did not have all the relevant knowledge about delivering cleft services needed to make funding decisions:

*“We had a board of people looking at things like the clinical service specification and stuff and the commissioners really were making the decisions, which was slightly worrying because you never felt that they knew the service that well”.* (CD 9)

*There is clearly a difference in opinion as to what was required to deliver cleft care and the commissioners then sort of went for the cheaper option – a lowest common denominator approach”.* (CD 5)

Funding was perceived by some Clinical Directors to be based on many historical ‘strategies’ that were not well understood rather than one standard, financially coherent model:

*“In addition to that the money is even more complicated because there is still existing historical parts of services which paid for under tariff, so, both [Location] [Location] and, are claiming through tariff. They are claiming for the operations that are done, they are claiming for the consultant lead outpatient appointments and things like that. But, they are still getting some money.”* (CD 4)

The ‘National Tariff’ approach funds teams either on an historical agreement or a ‘block’ contract based on the number of babies operated on per year as a package of care. However, this was not standardised across centres and some perceived that the tariff resulted in underfunding:

*“The problem has been that we were funded on a block contract of 80 new babies a year for years. Then this national tariff came in and because we didn’t have very savvy managers and as a Clinical Director I wasn’t savvy at all about the funding, …with the staffing level that we need to cover the network and the tariff only pays for episodes at the hub, so operations and a few of the cleft clinics. You know, we weren’t actually covering our costs at all. So for about two years we were grossly underfunded, and at the same time as I’m saying “I’d like another clinical psychologist please”.* (CD 6)

One team declared that cleft services were not comparable with the national tariff. They devised their own age-related tariff contingent across age bands and wanted to be paid agreed sums per band:

*“So separately [Organisation] decided to make it much simpler and say “well we have some intense periods of activity in the first three years, and then we move towards a more ‘speechy’ phase of their life from age 4 to say age 8. And then you move into an orthodontic and then the sort of final catch-up period. So they worked out that if you divided things up into three age bands I think it is, and then if you wrote down all the personnel that you’ve got in your team down the left hand side and asked them to kind of estimate what percentage of their time did they spend with each patient? So you know clinical nurse specialists aged 0–3, probably 80-90% of their time is spent on that group. You see what I mean, so you could do that for everyone and you can work out based on that kind of idea a tariff based on the big assumption that they actually already have the infrastructure to be CSAG compliant. And if you compare the provision for 60 or so babies a year compared with [Number] ours, we were woefully under. However if we got paid at the tariff as worked out that way, we could afford to be CSAG compliant. So we’ve managed to persuade our commissioners this year to pay us on an age band related tariff. It’s not a complete victory because what they’re saying is …“we’ll pay you on that basis until we actually get to the money we paid you last year”.* (CD 9)

Concerns were raised about funding on a cost per patient basis related to the numbers of operations performed. There were perceived inconsistencies in the way that procedures were recorded.

*“I have tried to change our commissioners’ focus explaining that more operations on any given patient are not necessarily an indicator of a high quality service and may in fact indicate a higher complication or revision rate. However, they do not seem receptive to these points. In fact, at one of our annual review meetings I was given a hard time by one of our commissioners who asked me why it was that [Location] had done “so many more” primary palate repairs than I had. Clearly, there appear to be differences in the way primary surgical procedures are recorded. Until there is consistency and accuracy in reporting these basic data I think it will be very difficult to make any meaningful comparisons between services or determine allocation of funding”.* (CD 5)

The Clinical Directors also acknowledged some regional differences in funding of cleft services that were not made transparent:

*“At the moment we’re funded, basically, on a block contract for 80 new babies a year; well we treated [Number] last year. And [Location] is also funded effectively on a block contract, I think they talk about being on a package of care … But either way I know, and the commissioners know, but no one’s actually said it in writing, that for the same baby in a different centre the funding is completely different”.* (CD 6)

This lack of a coherent model was deemed unfair by many Clinical Directors and they called for a more equitable approach.

*“The principle is that we need equity of cleft care and I’m very, very vociferous about this, so that’s kind of where we are – every centre has a different model of funding. Unbelievable.”* (CD 11)

There was also discussion about charges allocated within the National Services Definition set and how specific procedures were defined and recorded with reference to the chance of error in definition and recording of these procedures.

*“There’s this thing called the National Services Definition – there are two spreadsheets that you have to look at the same time - for a procedure and then there is a diagnostic code. And the assumption is if you have a diagnosis – you have a procedure that sounds like cleft that’s a cleft procedure on a cleft patient. The trouble is if you validate that there will be an error of about 20% either way. So you might have a dental extraction in your Max-fax clinic which has been charged as a cleft, when in fact it isn’t.”* (CD 6)

Eventually a Clinical Specification Document was developed that provides a basis for the provision of cleft services. It was adopted by the Specialist Commissioning Group as the standard for all cleft care.

### Capacity and budget allocation

Clinical Directors from services with more than one operative site felt that specialist commissioners were not overtly supportive of them and perceived that services were underfunded. Major concerns were limited resources and cuts made by the Department of Health that resulted in a struggle to provide an adequate service because of internal competition:

*“…. commissioners must be aware that the money they give to local trusts, a big part of that goes in overheads. Which means less and less money is available for frontline service. We have basically trimmed down we are now that lean and very soon we will be undernourished so to speak so that will have direct implications for the care we provide so that’s is a big issue that may be relevant for the national overall strategy because it may be multiplied in other centres.”* (CD 11)

Some Clinical Directors perceived a need to ‘ring fence’ money as profits from specific health care services were not always re-invested into that same service which could leave services under-funded:

*“Our arguments over funding are more with the Trust. The Trust will take any profit and leave you with the bare bones. They will not ring fence it. And it’s a problem really. It’s ok as long as we’ve got enough staff, but it’s when they start saying “right you’ve got to cut, you’ve got to cut” and we say “well hang on a minute, you’ve got loads of money coming in from us, why should we?”* (CD 9)

The child’s transition through the Cleft Treatment Pathway into adult services was considered to have been neglected in the centralisation process. Individuals born with a cleft may need to return to the service as adults or they may opt for interventions that they had refused when they were younger. Adults may change location and require cleft-related care from a different service, but funding does not necessarily follow them:

“….*nobody realised what a steady flow of adults would come back to us. Anything from 25 up to 70 or 80 and that does seem to be a reasonably steady flow. And then there are the kind of other referrals that we get so, as you probably heard us talk about, they’re, and all centres are finding that if you look at referrals that are not new baby referrals, I mean we run at about an average of 100 a year so it might be cleft patients coming in who’ve moved into the region”.* (CD 6)

### Impact on staff and staffing levels

Centralisation had a big impact on cleft service staff. Many had to re-apply for their posts and there was pressure from commissioners to use funds for appointments quickly; consequently, teams perceived that they were given insufficient time for planning:

*“The commissioners were telling us ‘and by the way we would like you to spend £[Number], on staff this year and we know we’re half way through the year but get on and spend it.’ And ‘What do you mean you haven’t spent it all?’ We were saying that we want to plan how we want to spend this money and then we’ve got the appointment process and actually the only people we can spend it on is our Clinical Director and Network Manager this year and we’ll be struggling to spend in on very many other people.* (CD 4)

The geographical diversity of sites also caused some difficulties, particularly with regard to staff travelling to and between cleft service centres:

*“… one thing that wasn’t thought through when in this whole concept of centralisation, be it clefts, is the impact on the staff. We have had a lot of resignations from people who couldn’t stick the travelling between the two centres…That’s my sort of take-home message as being somebody who’s been part of an MDT here. Travel will lead to burn out in most of the team members eventually. I think that is something has to be thought of very carefully.* (CD 7)

## Discussion

This qualitative case study explored views of cleft services post-centralisation. In-depth interviews were conducted with 11 Clinical Directors from U.K. Cleft Service. Findings show that Clinical Directors perceive the commissioning of cleft services in the U.K. to be inequitable as they are often dependent on historical agreements and individual negotiation with commissioning bodies. However, it is important to note that Clinical Directors’ opinions of and comparisons to cleft services other than their own may not be based on direct experience of these services.

### Strengths of the research

To our knowledge work of this type has not been carried out within the U.K. Cleft Service previously and we had a good response rate from Clinical Directors with 11 out of 14 agreeing to be interviewed. The research is timely in that it was undertaken when commissioning of cleft services was under scrutiny by the NHS commissioning body with particular regard to equity and excellence in service delivery. Despite using two different modes of interview (face to face and by telephone) we are not aware of any difference in the quality of the interviews. For example, one of the most 'abrupt', fastest, 'clinical' interviews was face to face, whilst one of the longest and ‘open’ was conducted on the telephone. Most Clinical Directors had met J.K.S. previously and so that any differences in interview content are considered to be a function of personality rather than methodology.

The ontological position taken in the analysis of data was ‘interpretative’ which posits that methods of natural science are not appropriate for social enquiry as the social world is not governed by regularities that hold law like properties [10]. However, a potential weakness of the study is that A.S. and J.K.S. were each aware that independent interpretation of the interview content by J.K.S and A.S. may have been influenced by their own individual perspectives and values. Multiple interpretations may occur as a function of the researchers’ own social experience, experience of the nature of the topic under investigation and professional role. However, with A.S. (an experienced qualitative researcher but with no experience of cleft services or commissioning) taking the lead in data analysis we endeavoured to optimise objectivity and reduce the possibility of multiple interpretation of the data as A.S., an experienced qualitative researcher who has not worked within the cleft service or had experience of commissioning in health services, took the lead in data analysis. Indeed, A.S. had no prior social or professional experience of the interviewee’s, or their opinions relating to the delivery of cleft services in the U.K. Furthermore, A.S. and J.K.S. discussed any discrepancies in the interpretation of the emerging themes until consensus was reached and therefore, the risk of potential bias arising as a function of J.K.S.’ role as a professional with a special interest in cleft was minimised.

### Funding and delivery of cleft services

Despite centralisation and the development of specialised services, Clinical Directors perceived unfairness in the commissioning and funding of cleft services. Reported inconsistencies in funding models and service costs have implications for delivering an equitable cleft service with an effective Multidisciplinary Team. A major concern was that funding was often based on numbers of cleft babies entering the service rather than the actual clinical procedures carried out. Funding based on a ‘numbers’ basis risks the reward of models of care in which multiple “revision” surgical procedures take place to rectify unsatisfactory outcomes or complications. This model does not account for the additional burden of care of non-cleft patients, adults and people transferring between units. Such ongoing needs and interventions are difficult to specify in advance and commissioning contracts may remain incomplete (although previous data could be used to estimate future funding needs). Non-standard needs also mean that it is harder for commissioners to judge whether appropriate services are provided [13].

In order to overcome such hurdles, it is important that accurate and appropriate data are available to support service provision. The CSAG report advocated cleft team participation in clinical audit and noted that data relating to all cleft patients and procedures should be available for comparative research. Audit data is shared at professional meetings such as the Craniofacial Society of Great Britain and Ireland (CFSGBI) annual meetings and could also be shared with commissioners in order to facilitate effective commissioning. The CRANE database records data about the provision of cleft services and provides an opportunity to further integrate and standardise cleft data. This too would benefit the commissioning of services via the identification of regional variation in procedures and outcomes [14].

### Consultation with families and satisfaction with outcomes

In the provision of any service it is important that all stakeholders have a voice. While Clinical Directors spoke of their commitment to family consultation in their pre-CSAG service scoping efforts, it is not clear to what extent parents’ voices are heard by commissioners. Regarding the provision of cleft care, families and individuals can voice opinions about cleft service provision through Healthwatch U.K. (http://www.healthwatch.co.uk), the Cleft Lip and Palate Association (CLAPA, http://www.clapa.org), Patient Reported Outcome Measures (PROMs) and via both national and regional cleft teams. However, if this information does not reach commissioners there is a danger that they may act on the basis of weak information about patients’ and parents’ preferences [15]. Cleft teams have direct contact with parents and could act as a conduit for the gathering of data about their perceived needs and opinions although they may not be as objective as an independent body. More objective data about parental views could be collected from the Cleft Lip and Palate Dashboard developed by the cleft Clinical Reference Group (CRG). This records Key Performance Indicators which might be used as a benchmark for setting predicted levels of service performance although it is important to note that PROMs are not currently included [16]. Data submitted to the Dashboard will ultimately be shared with both commissioners and the general public, with each centre’s results identifiable. Families and individuals can also voice their views of cleft service provision through Patient and Public Involvement (PPI) initiatives such as that supported by the Cleft Collective [17].

### Future of cleft services commissioning

At the time this study was conducted, commissioning for cleft services was complex, inequitable and confusing. The study highlights that good relationships and communication between teams and commissioners are essential for optimal provision of cleft services. For the last decade, commissioning of clinical services has been conducted by Primary Care Trusts. However, from April 2013, as a consequence of the Health and Social Care Act 2008, commissioning Primary Care Trusts ceased to exist and the specialised commissioning function was transferred to Clinical Reference Groups that cover all prescribed specialised services [18].

A new national model, aimed at improving service access, quality and efficiency, and reducing variation, is being developed by the NHS Commissioning Board. This will be led by ten Local Area Teams working closely with specialised health care providers and Centralised Commissioning Groups. This should ensure that all patients will have equal access to high quality services irrespective of where they live. Clinical Reference Groups have responsibility for preparing national specialised service strategy and developing specifications and policies. Clinical Reference Groups unite clinicians, commissioners, and Public Health experts with the patients and carers who use the relevant services. Members are volunteers with a particular interest, knowledge or experience in specialised healthcare [19]. Within this new framework it is proposed that Clinical Reference Groups work closely with specialist health care providers with a view to addressing capacity, funding and impact issues within the context of changes to commissioning services to ensure all patients have equal access to high quality services irrespective of where they live. Similar studies could then be undertaken to consider the views of commissioners, and service users and the implications of these individual views for policy and clinical practice in specialised services such as cleft.

## Conclusion

Clinical Directors’ accounts of their relationships with specialist commissioning bodies and their perspectives of funding cleft services may serve to increase parity and improve the commissioning of cleft services in the U.K. There is also scope for NHS commissioners to benefit from Clinical Directors’ accounts of their experiences of commissioning in order to work towards a new national model of cleft and other specialised care services. This could improve access to specialised services, quality and efficiency, and improve the experiences, not only of patients and carers, but also of health care providers working within these services.

## Abbreviations

CLP: Cleft lip and/or palate
CRG: Clinical reference group
NHS: National health service
CFSGBI: Craniofacial society of Great Britain and Ireland
CSAG: Clinical standards advisory group
MDT: Multidisciplinary team
PCT: Primary care trust
NDS: National definition set
SCG: Specialised commissioning group
SHA: Strategic health authority
NRES: National research ethics committee.
